# The Antecedents of Poor Doctor-Patient Relationship in Mobile Consultation: A Perspective from Computer-Mediated Communication

**DOI:** 10.3390/ijerph17072579

**Published:** 2020-04-09

**Authors:** Mengling Yan, Hongying Tan, Luxue Jia, Umair Akram

**Affiliations:** 1 School of Economics and Management, Beijing University of Posts and Telecommunications, Beijing 100876, China; emilyatpku@126.com (M.Y.); jialx2302@163.com (L.J.); 2 Guanghua School of Management, Peking University, Beijing 100871, China; akram.umair88@pku.edu.cn

**Keywords:** poor doctor-patient relationship, healthcare consultation, mobile context, computer-mediated communication

## Abstract

This study aims to understand the underlying reasons for poor doctor-patient relationships (DPR). While extant studies on antecedents of poor DPR mainly focus on the offline context and often adopt the patients’ perspective, this work focuses on the mobile context and take both doctors’ and mobile consultation users’ perspectives into consideration. To fulfill this purpose, we first construct a theoretical framework based on the Computer-Mediated Communication (CMC) literature. Then we coded 592 doctor-user communication records to validate and elaborate the proposed theoretical model. This work reveals that characteristics of mobile technologies pose potential challenges on both doctors’ and patients’ information providing, informative interpreting, and relationship maintaining behaviors, resulting in 10 and 6 types of inappropriate behaviors of doctors and users, respectively, that trigger poor DPR in the mobile context. The findings enrich the research on online DPR and provide insights for improving DPR in the mobile context.

## 1. Introduction

The emerging use of mobile medical consultation in China has propelled the establishment of doctor-patient relationships (DPRs) in the mobile context. DPR relies on mutual familiarity, trust, and interaction between physicians and patients during healthcare planning [1], and is essential for developing superior healthcare services. Given its significance, ample attention has been paid to exploring the antecedents and outcomes of DPR [1,2,3,4]. With the wide application of mobile medical consultation in China, people are allowed to interact with doctors to make inquiries and obtain medical information through computer-mediated communication [4,5]. Through this service, the scenes where DPR is established are extended from the offline context to the mobile context. In addition, mobile medical consultation service offers medical information not only for patients but also for other users who are not necessarily patients. To avoid confusion, we use the term “user(s)” when discussing DPR in a mobile context.

Compared to the rapid increase of mobile medical consultation users, perceptions towards mobile DPR is less optimistic. According to a related industry report, more than 40% of doctors have reported that they consider the DPR to be tense in a mobile context [6]. Hao and Zhang [7] found that 12% of users made negative comments on the treatment effect, and 9% made negative comments on the service attitude of doctors on a Chinese mobile consultation platform. Poor DPR not only impairs users’ health conditions at the individual level [8] but also causes serious social problems at the society level [9].

Extant research on mobile healthcare service is emerging, but studies that aim at uncovering the underlying reasons for poor DPRs in the mobile context are still lacking. The majority of previous research tends to focus on the positive experience brought about by mobile healthcare services, such as user satisfaction [10] and the adoption or continuous usage of mobile technology [3,11]. However, the experience of dissatisfied participants is largely ignored. The negative experience is also worth noting because understanding the complaints guide practitioners to improve service quality [8]. Although a few studies have paid attention to the dissatisfying experience of mobile healthcare services, they tend to interpret the experience only from the perspective of users [8,12]. While these studies are insightful, a single perspective from users is not adequate, because they missed the perception of doctors which is considered quite different from that of users [13,14].

These two literature gaps (namely lacking studies on dissatisfying experience of mobile healthcare services and lacking dual perspectives from both users and doctors) might partly be attributed to the mainstream research method in the healthcare field. Most studies rely on questionnaires and interviews to collect subjective ratings about mobile healthcare services, such as Akter et al. (2013) [15], Deng et al. (2015) [16], and Wu et al. (2018) [17]. The collected responses are usually inaccurate since respondents are rating events that happened at an earlier time. Besides, it is difficult to match responses from doctors and patients via questionnaires.

This work aims to uncover the underlying reasons for poor DPR from dual perspectives of both doctors and users in mobile medical consultation service. To achieve our goals, we first reviewed the literature on Computer-Mediated Communication (hereafter, CMC) in search of theoretical accounts for the poor DPR in the mobile context. The CMC literature focuses on the influence of the features of CMC on communication processes, which enables us to understand the potential negative impacts that CMC brings to doctor-user communication. As a result, the CMC literature guides us to identify the underlying reasons and mechanisms of poor DPR in the mobile context [18]. 

Next, we conducted an in-depth qualitative analysis based on objective communication records collected from a leading Chinese mobile healthcare application, Chunyu Doctor, to validate and refine the theoretical accounts. Chunyu Doctor is a commercial mobile consultation platform that connects users who search for medical information and doctors who work in public hospitals in China. On this platform, doctors are free to define their service prices and can earn legal income by providing consultation services for users. Meanwhile, users can pay a fee to consult doctors and make service evaluations after the consultation. Users can consult doctors either by telephone or by texts and pictures, but the latter is more frequently adopted in practice. This mobile platform is chosen due to the following two reasons. First, founded in 2011, Chunyu Doctor was among the first to start a mobile consultation service in China. By the end of 2017, it had accumulated 125 million users and 500 thousand physicians and conducted more than 330 thousand consultations per day, which allows us to get access to a large number of real communication records. Second, Chunyu Doctor provides users with a service evaluation system, in which a user can rate the service as “satisfied”, “general” or “dissatisfied”. Analyzing communication records rated as “dissatisfied” is helpful to discern potential problems in mobile consultation from both users’ and doctors’ perspectives.

The findings of this work contribute to the theorizing and understanding of DPR in the mobile context by offering theoretical accounts from the perspective of CMC. We also shed light on effective ways to improve users’ or doctors’ satisfaction towards mobile healthcare service. Both users and doctors are suggested to change their expectations and interaction habits to better adapt to the features of mobile communication.

## 2. Theoretical Background

To understand the key antecedents of poor DPR in the mobile context, we first reviewed the CMC literature to summarize the features of CMC and their potential negative impacts on communication. Then, we narrowed down our discussion on the relationships among CMC, doctor-user communication, and DPR in the mobile context, and proposed a theoretical framework that explains the antecedents of poor DPR in the mobile context.

### 2.1. Features of Computer-Mediated Communication

Computer-Mediated-Communication (CMC) refers to communication-based on computers and the internet, such as e-mail, web messaging systems, online forums, and mobile applications [19,20].

Abundant studies have examined the features and differences between traditional face-to-face communication and CMC [21]. Based on an in-depth literature review, we identified four features of CMC, namely connectivity, text-based communication, asynchronism, and anonymity. Connectivity refers to the fact that users can initiate or participate in online interaction regardless of time and space limits [22]. Text-based communication refers to the fact that the majority of communication is delivered through texts, lacking audio or visual clues [20]. The asynchronous nature of the media implies that there is a time delay during the communication [23,24]. Admittedly, as technologies keep upgrading, voice, picture messages and even synchronous video communication are also supported by CMC, but they are still used in relatively low frequency. Finally, anonymity refers to the fact that CMC enables users to hide his or her real identity by using a screen name, which is considered as the most remarkable difference between CMC and traditional offline communication.

CMC brings both positive and negative impacts on the communication process. In Table 1, we draw on extant studies and summarize the potential positive and negative impacts that CMC may have on users’ online communication behaviors. In this study, we apply CMC in the mobile medical consultation context and focus on the potential negative impacts. 

### 2.2. A Computer-Mediated Communication Perspective on Poor DPR

DPR refers to the collaborative and affective bond between doctors and patients [35]. Satisfaction has been proved to be a critical determinant of DPR [2,36]. For the patient, patient satisfaction significantly increases the likelihood of the patient returning to the doctor for treatment. If the patient’s needs are met during the service, there will be fewer complaints and medical disputes, which contributes to positive DPR [4]. For the doctor, doctor satisfaction can increase doctors’ work enthusiasm and promote the willingness to establish a friendly relationship with patients [36]. In summary, satisfaction is a key driver for improving DPR for both doctors and patients. Accordingly, unsatisfactory service experience will lead to poor DPR for both doctors and patients [14].

Effective doctor-patient communication is essential to realize satisfactory service and maintain harmonious DPR [5,37]. On the contrary, undesirable doctor-user communication can cause poor DPR [14]. Extant studies consensus on the use of informational and emotional dimensions to depict the communication processes between doctors and patients [1,17,38]. Informational-oriented communication, also termed as task-focused communication [38,39], refers to communication on medical information provision and interpretation. To be more specific, the informational communication can be divided into information providing and information interpreting [14,40]. Emotional-oriented communication, also termed as socio-emotion-focused communication [38,41], refers to communication on the identification and response of emotional cues. Emotional-oriented communication is conducive to meeting both doctors’ and users’ emotional needs and maintaining a friendly relationship [41]. Both informational-oriented and emotional-oriented communication are two-way communications between doctors and users.

When the medical environment shifts from the traditional face-to-face context to the mobile context, the features of the medium that supports doctor-patient communication have also changed [18,42,43]. While traditional face-to-face medical communication relies on synchronous communication with language tones and facial or body cues, mobile communication relies on text-based asynchronous communication [18]. According to media synchronicity theory, features of media determine the media capabilities in supporting information transmission and information processing and further determine the communication outcomes [40]. Therefore, there are reasons to believe that the features of CMC will impact the doctor-user communication process and further impact DPR in mobile consultation.

**Figure 1:**
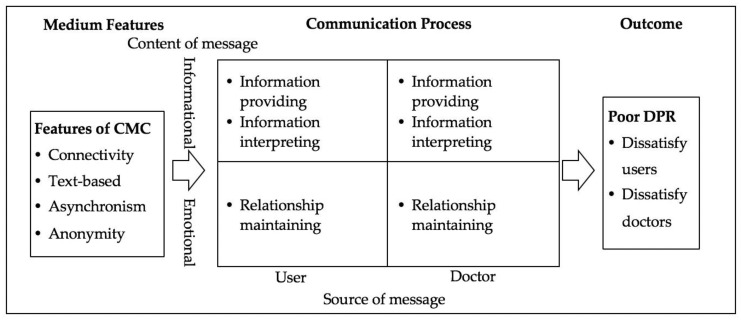
Theoretical framework.

Based on the above arguments, we propose a theoretical model (as is shown in Figure 1), aiming at explaining the antecedents of poor DPR in the mobile context. The key arguments of this model are: (1) Features of CMC create barriers for information providing, information interpreting and relationship maintaining for both doctors and users during the two-way communication, and (2) the undesirable doctor-user communication caused by features of CMC leads to poor DPR that is manifested by doctors’ and users’ dissatisfaction.

While this preliminary framework sheds light upon the logical relationships between CMC features, doctor-patient communication, and DPR, it also reveals several directions for further exploration: 1) it is unclear what representative information providing, information interpreting, and relationship maintaining behaviors of doctors and users lead to poor DPR, and 2) it is unclear how limitations of CMC account for these behaviors. As a result, this preliminary theoretical framework provides initial answers to our research question and guides our data analysis to answer the remaining questions. 

## 3. Research Method

**Figure 2:**
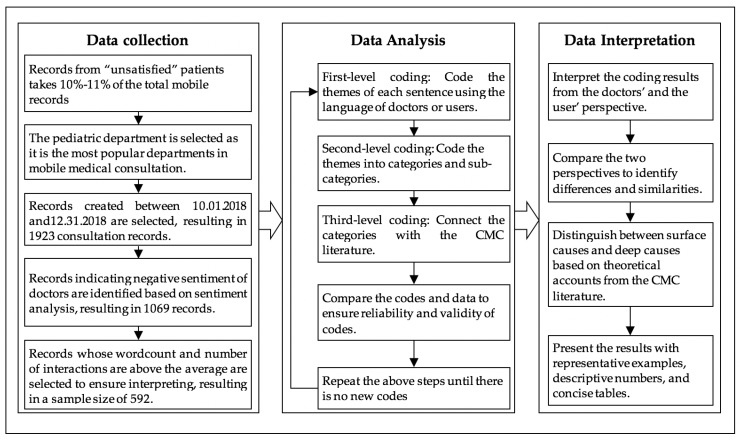
The methodology roadmap.

To empirically validate and elaborate our proposed theoretical framework, this work employs netnography, or internet-based ethnography, as the qualitative research method [41,44,45]. The study proceeded in three steps: (1) developing a preliminary coding plan based on the CMC literature (as is shown in Figure 1), (2) downloading and coding objective communication records as well as comments that are rated as “dissatisfied” by users in the selected mobile application; and (3) analyzing the data to identify representative interaction behaviors from users’ and doctors’ perspectives. The detailed steps of data collection, data analysis, and data interpretation are shown in Figure 2. 

### 3.1. Data Collection

Mobile consultation service allows users to chat with professional and experienced doctors in real-time by sending messages with texts and photos. The electronic medical records of patient-doctor communication during the online consultation process are mainly text-based. Therefore, communication records between doctors and users are valuable materials that worth analyzing. Researchers can analyze these communication records from the perspectives of both users and doctors, and gain an insight into the online patient-doctor communication process.

The communication records used in this study were collected from Chunyu Doctor. We analyzed the communication records that were rated as “dissatisfied” by users on the platform. A Java-based program was developed to automatically download the communication records between doctors and users. On average, consultation records that are labeled “dissatisfied” by users take 10%-11% of the total records. In total, we have downloaded 1923 “dissatisfied” interaction threads between 633 doctors and 1923 users from October 1st, 2018 to December 31st, 2018 in the pediatric department. The period was selected because this quarter of the year is reported to have the highest average monthly user activity in Chunyu Doctor [46]. The pediatric department was selected due to two reasons. On the one hand, pediatrics is the most frequently visited department in mobile consultation due to the shortage of pediatricians in offline hospitals. On the other hand, collecting data from pediatric is conducive to reflecting poor DPR in mobile consultation, because users in the pediatric department are usually the guardians of patients rather than patients themselves. And guardians who have strong feelings for their loved children are more likely to have conflicts with doctors [47]. The “dissatisfied” consultation records represent users’ dissatisfactory experience. Additional steps were taken to screen records that reflect doctors’ dissatisfactory experience. To be more specific, we used a widely applied Python-based program of Chinese sentiment analysis to obtain the sentiment score of all words generated by the doctor in each dialog. The accuracy of this program is tested as 0.8277 [48]. Through the analysis, results show that the mean value and the variance of doctors’ sentiment in 1923 records are -0.0642 and 0.1645 respectively. Records that score in the range of -1 to 0 indicate potential negative emotions of doctors. Based on this analysis, the research team manually went through all the selected records to ensure accurate identification, resulting in a sample size of 1069 records. Finally, to rule out the possibility that poor DPR is a result of insufficient communication, we selected communication records with word counts and the number of interactions during the communication above the average. As a result, a total of 592 detailed consultation threads from 358 doctors were collected for analysis. In the final sample, the total Chinese characters amount to 166,985, the average word count is 282.07, an average number of interactions for each communication thread is 33.24.

### 3.2. Data Analysis

To analyze the text-based communication records, qualitative analysis is considered appropriate [12,14,41,49,50]. Specifically, using netnography and coding skills from the qualitative analysis [51], the qualitative data analysis proceeded in the following four steps. 

First, first-level coding. This is also referred to as open coding in classic qualitative analysis, where topics are generated from words or sentences of the original material [51]. Researchers of this study coded each line of communication records as well as user’ comments after the consultation experience using the language of doctors or users. To ensure the validity and reliability of qualitative coding, three researchers read and coded the original communication records independently. After each of their initial coding was completed, they go through all the coding results and discuss different opinions through in-depth discussion until they reached consensus. 

Second, second-level coding. This is also referred to as axial coding in classic qualitative analysis, where topics are consolidated and abstracted to categories and sub-categories based on comparison and contrast [51]. Usually, the categories and sub-categories may appropriate the terms and phrases from the literature. As a result, first-level codes in our study were further classified into informational and emotional dimensions. Through this step, doctors’ information-related behaviors and emotion-related behaviors that cause users’ dissatisfaction, as well as users’ information-related behaviors and emotion-related behaviors that cause doctors’ dissatisfaction, are obtained. 

Third, Third-level coding. This is also referred to as selected coding in classic qualitative analysis, where categories are connected to tell a logical story of the intended phenomenon [51]. We counted the frequencies of each identified category and selected categories with high frequencies to form the complete model that explains the antecedents of poor DPR in the mobile context. Based on these selected categories, challenges of mobile technologies identified using the CMC literature and interaction behaviors of doctors and users identified in the communication records are connected. Specifically, each of the researchers tried to understand the underlying reasons behind doctors’ and users’ mobile misbehaviors by referring to the CMC features identified by the CMC literature. To ensure the validity and reliability of the classification, three researchers conduct this step independently and converge opinions through in-depth discussion. 

Forth, developing coding schemes. Based on the above three steps, we developed a coding schemes, and use this coding scheme to code subsequent consultation records. To ensure the reliability and validity of the codes, different researchers repeat the above coding steps and compare the codes and data to reach a converged opinion. The above coding steps repeat until there are no new themes, categories, or sub-categories that are generated to explain the original data.

## 4. Findings

### 4.1. The Users’ Perspective

By adopting the users’ perspective, we identified 10 representative types of doctor behaviors, as is shown in Table 2. In the following, we introduce quantitative coding results and representative behaviors of doctors in terms of the information providing, information interpreting, and relationship maintaining categories.

#### 4.1.1. Barriers for Doctors in Information Providing

That doctors fail to provide accurate and adequate medical information for users over mobile consultation is a major reason that causes users’ dissatisfaction. Nowadays, users of mobile medical services require richer information to understand their physical conditions and make reasonable medical decisions [7]. They not only require information about the treatment suggestions, but also require information about why they become sick, how to treat the disease, and why the doctor makes a specific diagnosis. However, in the mobile context, text-based and asynchronous communication makes it more difficult for doctors to reply to every message from users. Specifically, doctors may fail to explain etiology, diagnostic evidence, and fail to offer clear and effective suggestions, which leaves users’ information-related needs unmet. As a result, users complain during their communication with doctors or in their service comments, such as “the doctor didn’t explain the causes of my disease”, “I’m not clear about how he makes this diagnosis”, or “the advice is not detailed enough”. Of all our codes for the doctors’ behaviors from the users’ perspective, the percentages of codes indicating lacking etiology analysis, lacking diagnostic evidence, lacking operational advice and ambiguous answers account for 6.42%, 18.92%, 16.22% and 25.00% of the dissatisfying conversations respectively. 

Here we provide an example for lacking etiology analysis. A user consulted a doctor on the causes of his child’s symptoms. The doctor diagnosed the symptoms as viral infections and gave drug recommendations. The user inquired again about the etiology, but the doctor ignored the user’s question and gave advice on medication again. The user then complained, “I’m asking you about the causes of the disease, doctor. You only tell me what medicine to eat”. 

Users also frequently complain about a lack of diagnostic evidence during mobile consultation. Users sometimes ask their doctors, “how did you make your judgment?”, “why did you choose this medicine over that one?”, or “how did you come to the treatment plan?’. The doctor usually repeated his suggestions, ignored users’ questions, or replied “it is too complex to explain to you”. 

Lacking actionable advice is another type of behavior frequently complained by the users. Sometimes, due to limited diagnostic clues or mild symptoms, doctors may suggest continuous observation without any actionable advice. Many users find this suggestion unacceptable and evaluate the mobile consultation service as dissatisfactory. Actionable advice creates a sense of security because users feel they can do something to prevent the disease from worsening [16]. Moreover, actionable advice is consistent with users’ offline consultation expectations.Most users decide to go to offline hospitals only when they have severe symptoms. As a result, most users get actionable advice from their doctors [52]. When users extend their offline expectations to the mobile consultation service, lacking actionable advice may easily cause dissatisfaction. 

Ambiguous answers may cause dissatisfaction of users. Doctors sometimes provide general rather than customized suggestions to users due to limited diagnostic clues, time constraints, or simply because they ignore the specific requirements of users. An example of an ambiguous answer is shown in the following. A user described the symptoms of his child to a doctor, and the doctor answered, “that may be bacterial infection”. Then the user asked again, “what are the causes?”. The doctor said, “not sure. Many factors can cause infection”. The user made a negative comment and complained “Too vague! The doctor didn’t give explicit answers”.

#### 4.1.2. Barriers for Doctors in Information Interpreting

In mobile consultation, doctors sometimes overlook information provided by users or provide irrelevant answers to users’ questions. This is in part because the asynchronism feature of CMC increases the difficulties of reading and interpreting information during the consultation. As a result, users feel their needs are neglected. Of all our codes for the doctors’ behaviors from the users’ perspective, the percentages of codes indicating doctors’ ignoring information provided by users and irrelevant answers are 3.04% and 3.38% respectively.

A typical example showing doctors ignore information provided by users is described in the following. A user told the doctor that his child had allergic rhinitis last year and then he described the symptoms that the child had. The doctor replied, “it must be the symptoms of rhinitis”. The user complained that the doctor only repeated the information provided in his symptom description.

Doctors sometimes provide irrelevant answers because they fail to understand users’ intentions. Here we provide an example for this case. A user asked his doctor “what is the harm of low fever?”. The doctor skipped this question and constantly asked “what is the temperature? Are there any symptoms?” As a result, the user complained, “I just want to know the harm of low fever. Why not answer this question straightway?”.

#### 4.1.3. Barriers for Doctors in Relationship Maintaining

Representative behaviors that fail to meet users’ emotional needs include delayed response, lack of initiatives, lack of emotional comfort, and being unfriendly, each takes 26.69%, 10.81%, 5.74%, and 13.18% respectively in our coding for the doctors’ behaviors from the users’ perspective.

During mobile consultation, doctors’ delayed response is a prominent issue that causes dissatisfaction of users. Asynchronous communication makes it difficult to guarantee the timeliness of doctors’ replies in mobile consultation. If doctors fail to respond to users’ questions promptly, users will feel neglected, disrespected and are not willing to establish a good relationship with their doctors [14]. Our data analysis reveals that when the mobile conversation is temporarily stopped due to doctors’ delayed response, users may complain, “I’ve waited for so long”, “I am very worried, can you hurry up to reply my questions”, or “your response is too slow”. 

Doctors are coded as “lack of initiatives” when they fail to provide additional information that is usually closely related to the questions asked by their users. For example, a doctor asked about what medicine the user was currently taking. After receiving the users’ reply, the doctor typed, “this medicine contains suspected carcinogen”. Since the doctor failed to provide choices or precautions during the conversation, the user felt very anxious and rated “dissatisfied” in the end. Typical complaints from users include “the doctor merely answers the questions that I ask, but not provide any additional information”, “cherish your words like gold”, or “the doctor talks as squeezing the toothpaste”. 

Lacking emotional comfort is also a common phenomenon that causes dissatisfaction of users in mobile consultation. Doctors’ emotional comfort has a strong effect on alleviating users’ negative emotions [41]. However, due to the lack of visual and auditory cues, doctors cannot effectively perceive the negative emotions of users and omit to express emotional comfort. For example, a user expressed his worry and anxiety about his child’s condition, but the doctor neglected the negative emotion and just put forward another question as a response. The user made a negative comment, “professional, but also so indifferent”.

Negative comments about doctors’ unfriendly attitude frequently happened. During mobile communication, doctors may use a strong tone or words, express the impatient mood, or use rhetorical questions, which makes users feel uncomfortable. As a result, users may complain like “too fierce”, “bad service attitude”, “not friendly at all”. Here we provide an example. When a user asked the doctor a question that he did not understand, the doctor replied, “I don’t need to repeat the question that I have explained! Haven’t I made myself clear?”. The user complained that “that’s terrible. I just ask a question, while he answers me like a teacher teaches a student”.

### 4.2. The Doctors’ Perspective

By adopting the doctors’ perspective, we identified 6 representative types of user behaviors, as is shown in Table 3. In the following, we introduce the results of quantitative coding and the representative information providing, information interpreting, and relationship maintaining behaviors of users.

#### 4.2.1. Barriers for Users in Information Providing

Users’ failure to provide accurate or adequate medical information to their doctors through mobile consultation is a common cause of doctors’ dissatisfaction. In traditional offline consultations, doctors acquire diagnostic clues through observation and examination. But in mobile consultations, medical clues, such as symptoms, prior medical treatment experience, and medicine usage, can only be provided by the users. However, due to the lack of professional knowledge, it is difficult for users to select useful medical information for doctors. Sometimes, they even provide a conflicting description or refuse to provide the information to assist the diagnosis. As a result, doctors complain during mobile consultation services, such as “why not answer my questions”, “no picture to assist my judgment”, or “I can’t understand your description”. Of all our codes for the users’ behavior from the doctors’ perspective, the percentage of codes indicating inadequate and vague diagnostic clues is 6.76%. 

Here we provide an example of failing to provide adequate clues. A user consulted a doctor on the causes of his child’s symptoms. The doctor replied, “it is hard to say, I can tell you based on a laboratory test, but it is hard to judge by naked eyes”. The user complained, “You are telling me nothing”. The doctors replied, “you did not even provide me a picture, you just keep sending me questions”. 

#### 4.2.2. Barriers for Users in Information Interpreting

During mobile consultations, users sometimes fail to understand the questions or suggestions offered by their doctors because they lack adequate medical knowledge or hold divergent medical opinions with their doctors. As a result, even though doctors spend much time repeating their opinions or explaining medical principles, users misunderstand their doctors’ suggestions. Of all our codes for the users’ behavior from the doctors’ perspective, the percentages of codes indicating users’ lacking adequate medical knowledge and holding conflicting opinions with their doctors are 4.39% and 1.01% respectively.

Here is an example that lacking adequate medical knowledge causes poor DPR. A doctor recommended formula milk to a mother because her child was diagnosed with milk protein allergy. Formula milk was suggested since it is easier for a child with dyspepsia to digest. However, this recommendation stimulated a strong objection from the mother. She said, “why not feed him with breast milk? He can’t get better with the formula milk. Are you kidding me!” Although the doctor had repeated the detailed medical principle to the mother, she still posted a negative evaluation of the doctor’s service. The doctor replied, “as a doctor, I recommend based on my knowledge and the condition of my patients”.

Information interpretation issues caused by conflicting opinions also appear in our codes. Some users stick to their inherent opinions that are formed in their prior experience [52]. This “confidence” sometimes leads to users’ difficulties in information interpretation. Conflicting opinions between traditional Chinese medical science and western medical science sometimes caused poor DPR. For example, the doctor made a diagnosis with western medical science, while the user tried to interpret the result from the perspective of Chinese medical knowledge. A user asked, “is that caused by the coldness of the body?” The doctor answered, “we are not talking about the same thing”. This is special in the Chinese context where traditional Chinese medical science co-exists with western medical science. Another example is the conflicting opinions about the treatment. For example, the doctor offered a treatment plan, but the user thought that taking medicine had unavoidable harm and asked for more conservative treatment. The doctor answered, “this treatment is necessary and you should follow doctors’ advice.”

#### 4.2.3. Barriers for Users in Relationship maintaining

During mobile consultation, users sometimes doubt the information offered by doctors, causing tense DPR from the doctors’ perspective. Users in the mobile era no longer rely on information from a single doctor. Instead, they search the internet, consult other doctors, and compare the information they collected from multiple sources and multiple times. Once there is conflicting information, the users tend to explicitly express their distrust towards their doctors during doctor-user communication. For example, users said, “I disagree with you”, “you might be wrong, I consulted multiple doctors and received conflicting recommendations”, or “no, I hear that… while you said that…”. In response, some doctors said during the interaction, “why do you distrust my opinion?”. Of all our codes for the users’ behavior from the doctors’ perspective, the percentage of codes indicating that users doubt the information provided by their doctors is 13.51%.

Sometimes, when there are divergent opinions, users even doubt the identity of their doctors. Different from the traditional offline context, the sense of authority and security used to associate with doctors is weakened in the mobile context. Moreover, users have less tolerance and understanding when their doctors make mistakes. In our codes, some users wrote, “I doubt whether you are a registered doctor”, “are you a real doctor/ an intern/a robot”, or “you’re not professional, I know it better than you”. As a result, doctors replied to these comments like “I’ve been working for 5 years”, “interns are not allowed to register for this service”, or “you are too rude”. Of all our codes for the users’ behaviors from the doctors’ perspective, the percentage of codes indicating that users doubt the identity of doctors is 3.38%.

In more extreme cases, users vent their negative emotions by giving personal remarks towards their doctors. For example, some users wrote, “I think you are a quack” or “why don’t you go elsewhere and sell your quack medicine to others”. As a result, during the interaction with these users, doctors replied by writing “please mind your tone”, “please do not use terrible words”, “show some respect”, or “you show no respect to me”. Of all our codes for the users’ behaviors from the doctors’ perspective, the percentage of codes indicating that users give personal remarks towards their doctors is 11.82%.

## 5. Discussion

**Figure 3:**
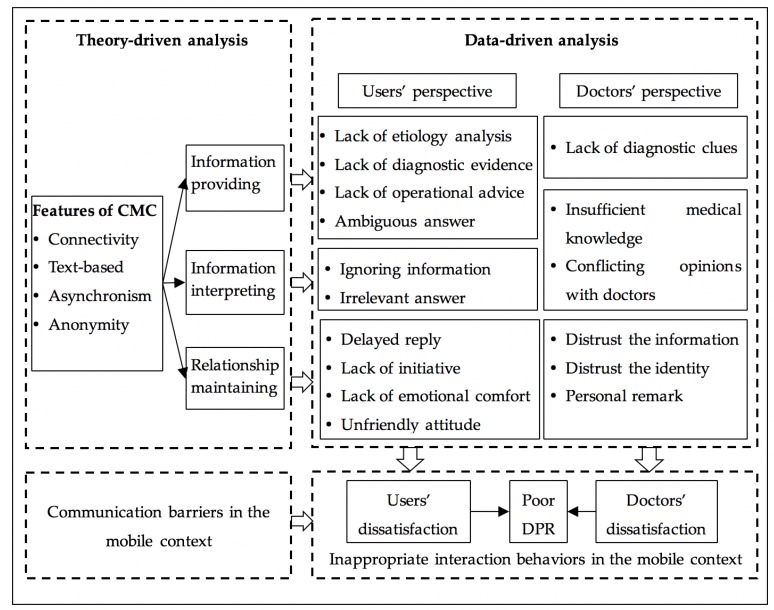
Antecedents of poor DPR in the mobile medical context.

This study discusses the impact of mobile technologies on DPR and pays special attention to the antecedents of poor DPR during mobile medical consultation. Figure 3 summarizes our key findings. 

First, inappropriate information providing, information interpreting and relationship maintaining behaviors of doctors and users are the direct causes of poor DPR in mobile consultation. From the perspective of users, mobile technologies have the potential to empower users with more medical knowledge and greater decision power over their health conditions [12]. However, their doctors fail to provide adequate support to realize the potential, which leads to user dissatisfaction and poor DPR. In specific, we find that some doctors fail to provide the etiology analysis, diagnostic basis, clear operational advice, or targeted answers to users’ questions. Moreover, some doctors ignore users’ emotional needs during communication and fail to provide a timely reply, an active inquiry, emotional comfort and/or a friendly service attitude to their worried users. From the doctors’ perspective, we find that some users fail to provide adequate or accurate diagnostic clues for their doctors, and some others fail to interpret the advice correctly due to limited medical knowledge or conflicting medical opinions. Moreover, we highlight that the emotional needs of doctors have been overlooked during doctor-user communication. Although doctors expect trust, respect, and understanding from users [18], they are susceptible to doubts and even personal remarks from users. These inappropriate communication behaviors directly lead to the dissatisfaction of doctors and users in mobile consultation services.

Second, doctor-user communication is compromised by CMC, which is the underlying cause of poor DPR in mobile consultation. The connectivity feature of mobile applications might lead to increased workload for doctors or information conflicting and overloading for users. Accordingly, for doctors, an overwhelming amount of workload may reduce the amount of time that doctors spend on each user, which may result in inadequate information providing and information interpreting. Meanwhile, users are more likely to be exposed to conflicting medical information, which might weaken their trust towards their doctors [14].

Features of text-based communication and asynchronism create barriers in medical information providing, information interpreting, and relationship maintaining behaviors for both users and doctors. For users, it is difficult to provide sufficient diagnostic clues to their doctors via texts and pictures. Moreover, lacking visual clues of doctors may weaken users’ trust toward their doctors. For doctors, lacking visual clues and asynchronous communication increase the difficulty to diagnose and interpret users’ symptoms, increase the time cost to provide a medical suggestion and increase the difficulty of perceiving users’ emotion [33,34]. 

The misbehaviors of users along the relationship maintaining dimension can be partly explained by the anonymity nature of mobile communication. In anonymous communication, people are more inclined to express negative emotions towards others compared to face-to-face communication because of reduced social presence [53]. Therefore, in mobile consultation users are more likely to an overt and explicit expression of negative emotions, which leads to poor DPR.

### 5.1. Theoretical Implications

Our findings have the following three theoretical implications:

First, this study extends existing studies on poor DPR by integrating both doctors’ and users’ perspectives. The majority of existing studies tend to investigate DPR only from users’ perspectives, such as Um et al. (2018) [8] and Zhang et al. (2018) [12], ignoring the significance of interpreting DPR from dual perspectives of users and doctors. Mobile consultation transforms the traditional doctor-dominated relationship to a more equal and reciprocal relationship [36,54], which emphasizes the importance of taking doctors’ experience into account to understand DPR. By discerning causes of poor DPR for both doctors and users, this study provides a more comprehensive understanding of DPR in the mobile context. 

Second, this research contributes to existing knowledge on the causes of poor DPR in the mobile context by elaborating on both direct and underlying causes of poor DPR. Existing studies mainly ascribe poor DPR to observed behaviors, such as long waiting hours, no treatment plan and impatience [7,14], which fails to explore the underlying reasons for these behaviors. By drawing on the theoretical perspective of CMC and by conducting a qualitative study on a leading Chinese mobile medical platform, this study not only highlights representative misbehaviors of doctors and users as the direct causes of poor DPR but also identifies CMC limitations as the underlying reasons. 

Last but not least, by comparing the traditional face-to-face consultation with CMC medical communication, this study extends the current mobile health studies by identifying the unique but dark side of mobile health services. Even though mobile health services are becoming extensively popular in recent years, the unique settings of mobile medical consultation make it difficult to further improve user satisfaction. By distinguishing the potential challenges of the mobile healthcare services, this study provides a brand-new perspective to explain user satisfaction, that is to explain user satisfaction from the mobile context itself, rather than from the interaction process. 

### 5.2. Practical Implications

Our findings also provide practical implications for doctors, users and mobile consultation application developers. Doctors are suggested to provide more support for users to take part in their medical decisions. In specific, besides diagnostic results, more and more users regard the diagnostic process, evidence, advice, and etiology as additional information to make a medical decision. When doctors overlook or refuse to follow up on these information needs, users often, but not always, feel dissatisfied. In the meanwhile, doctors are suggested to understand and respond to users’ emotional needs in mobile consultation service. For example, doctors should try their best to guarantee timely responses, make explanations or ask for understanding when responses have been delayed. 

Users are suggested to acknowledge the limitation of mobile consultation service, and adjust their behaviors and expectation to cope with the potential challenges brought by mobile-mediated doctor-user communication. To be more specific, users should offer adequate, accurate, and relevant medical information on their initiatives to help doctors to understand their physical conditions and emotional needs. Moreover, expressing understanding and respect is helpful for users to build friendly relationships with their doctors. 

Mobile consultation applications developers are supposed to optimize product designs by developing more effective tools to facilitate effective communication between doctors and users. For example, provide a template to instruct users to provide the required information for the diagnosis such as symptoms, examination reports, and medication use. The list of the required information help doctors collect users’ information conveniently and avoid repeated inquiries. Response templates are also helpful for doctors to provide detailed and standard medical information. Other useful implications for developers may include monitoring users’ waiting time during consultations to avoid users’ negative feelings and giving appropriate reminders to physicians when necessary. 

This study has several limitations, but it also points out several future directions. First, it is hard to capture doctors’ feelings and opinions in communication records. Although we adopt measures to screen records that can reflect doctors’ negative emotions, more effective methods of reflecting doctors’ feelings and evaluations are needed. Future studies are encouraged to use questionnaires or interviews to collect data from doctors, to supplement existing qualitative second-hand data and further discover potential problems from the doctors’ perspective. Second, to simplify the data analysis process, this study restricts doctor-user communication in one consultation. DPR stems from a long-term experience of care and counseling. Future research can consider the evolving characteristics of DPR and uncover the evolutionary process of poor DPR in the mobile context.

## 6. Conclusions

Mobile healthcare service has substantially changed the way people obtain medical service. Nevertheless, the majority of extant studies have focused on the potential positive impact and paid limited attention to potential challenges. We can refine the mobile consultation service and take better advantage of mobile technology by focusing on these potential challenges. This study uncovers the underlying reasons for poor DPR in mobile healthcare consultation services by taking both doctors’ and users’ perspectives into account. Drawing on the CMC literature, we identified the potential challenges brought by mobile technologies on the information providing, information interpreting, and relationship maintaining processes during doctor-user communication. Meanwhile, by analyzing the objective mobile communication records between doctors and users with qualitative methods, we identified representative misbehaviors of both doctors and users that cause poor DPR in the mobile context. We conclude that doctors and users’ inappropriate behaviors are the direct causes of poor DPR and limitations of mobile communication are the primary underlying cause of poor DPR in mobile consultation.

## Figures and Tables

**Table 1:** The potential positive and negative impacts of each CMC feature on online activities.

CMC Features	Potential Positive Impacts	Potential Negative Impacts
Connectivity	Transcend traditional time and space limitation, provide easy access to online information [25,26]	Increased workload for doctors [18] Information overloading [27] Conflicting information [14]
Text-based communication	Easy documentation and clear description of symptoms and instructions [23]	Higher-level cognitive effort compared to audio and visual communication [28] Lack of intimacy and weak at relationship development [20,29]
Asynchronism	Transcend traditional time and space limitation [25] Less interrupting [18] Flexible thinking time [30]	Hard to ensure timely response [31] Cause misunderstanding
Anonymity	Encourage expression [32,33]	Easy engender negative emotion and misconduct online [34]

**Table 2:** Antecedents of poor DPR from the users’ perspective.

Category	Subcategory	Percentage	Description
Barriers in information providing	Lack of etiology analysis	6.42%	The doctor fails to explain the etiology for the user.
	Lack of diagnostic evidence	18.92%	The doctor fails to provide the user with sufficient evidence for diagnosis.
	Lack of operational advice	16.22%	The doctor fails to give the user explicit operational instructions.
	Ambiguous answer	25.00%	The doctor’s answer is ambiguous.
Barriers in information interpreting	Ignoring information	3.04%	The doctor ignores the information provided by the user, such as symptoms.
	Irrelevant answer	3.38%	The answer provided by the doctor cannot answer the question asked by the user.
Barriers in relationship maintaining	Delayed reply	26.69%	The doctor is not able to respond to the user in time.
	Lack of initiative	10.81%	The doctor fails to provide relevant information actively.
	Lack of emotional comport	5.74%	The doctor lacks emotional comfort for the user.
	Unfriendly attitude	13.18%	The doctor is impatient and unfriendly

**Table 3:** Antecedents of poor DPR from the doctors’ perspective.

Category	Subcategory	Percentage	Description
Barriers in information providing	Lack of diagnostic clues	6.76%	The user fails to provide adequate or accurate diagnostic clues for the doctor
Barriers in information interpreting	Insufficient medical knowledge	4.39%	The medical knowledge of the user is inadequate to interpret or understand the suggestions from the doctor
	Conflicting opinions	1.01%	The user has difficulty in understanding the doctor’s advice due to different opinions with their doctors.
Barriers in relationship maintaining	Distrust towards the information	13.51%	The user doubts the correctness and reliability of the information provided by the doctor.
	Distrust towards the identity	3.38%	The user doubts whether the doctor’s identity is real or authorized
	Personal remark	11.82%	The user expresses dissatisfaction in a bad tone

## References

[1] Liu C.-F., Tsai Y.-C., Jang F.-L. (2013). Patients’ Acceptance towards a Web-Based Personal Health Record System: An Empirical Study in Taiwan. Int. J. Environ. Res. Public Health.

[2] Shaw B.R., Han J.Y., Hawkins R.P., James S., McTavish F., Gustafson D.H. (2017). Doctor–patient relationship as motivation and outcome: Examining uses of an interactive cancer communication system. Int. J. Med. Inform..

[3] Tang Y., Yang Y.-T., Shao Y.-F. (2019). Acceptance of Online Medical Websites: An Empirical Study in China. Int J. Environ. Res. Public Health.

[4] Liang C., Gu D., Tao F., Jain H.K., Zhao Y., Ding B. (2017). Influence of mechanism of patient-accessible hospital information system implementation on doctor–patient relationships: A service fairness perspective. Inf. Manag..

[5] Zhang X., Guo X., Lai K.H., Yi W. (2019). How does online interactional unfairness matter for patient–doctor relationship quality in online health consultation? The contingencies of professional seniority and disease severity. Eur. J. Inf. Syst..

[6] iResearch (2016). Report on the Demand of Doctors in China’s Online Medical Market. http://report.iresearch.cn/report/201609/2650.shtml.

[7] Hao H., Zhang K. (2016). The voice of Chinese health consumers: A text mining approach to web-based physician reviews. J. Med. Internet Res..

[8] Um K.H., Lau A.K.W. (2018). Healthcare service failure: How dissatisfied patients respond to poor service quality. Int. J. Oper. Prod. Manag..

[9] Zhou M., Zhao L., Campy K., Wang S. (2017). Changing of China’s health policy and Doctor–Patient relationship. Health Policy Technol..

[10] Gu D., Yang X., Li X., Jain H.K., Liang C. (2018). Understanding the Role of Mobile Internet-Based Health Services on Patient Satisfaction and Word-of-Mouth. Int. J. Environ. Res. Public Health.

[11] Mein Goh J., Gao G., Agarwal R. (2016). The creation of social value: Can an online health community reduce rural–urban health disparities?. MIS Q..

[12] Zhang W., Deng Z., Hong Z., Evans R.D., Ma J., Zhang H. (2018). Unhappy Patients Are Not Alike: Content Analysis of the Negative Comments from China’s Good Doctor Website. J. Med. Internet Res..

[13] Sewitch M.J., Abrahamowicz M., Dobkin P.L., Tamblyn R. (2003). Measuring Differences Between Patients’ and Physicians’ Health Perceptions: The Patient–Physician Discordance Scale. J. Behav. Med..

[14] Atanasova S., Kamin T., Petri G. (2018). The benefits and challenges of online professional-patient interaction: Comparing views between users and health professional moderators in an online health community. Comput. Human Behav..

[15] Akter S., D’Ambra J., Ray P. (2013). Development and validation of an instrument to measure user perceived service quality of mHealth. Inf. Manag..

[16] Deng Z., Liu S., Hinz O. (2015). The health information seeking and usage behavior intention of Chinese consumers through mobile phones. Inf. Technol. People.

[17] Wu T., Deng Z., Zhang D., Buchanan P.R., Zha D., Wang R. (2018). Seeking and using intention of health information from doctors in social media: The effect of doctor-consumer interaction. Int. J. Med. Inform..

[18] Lee S.A., Zuercher R.J. (2017). A current review of doctor–patient computer-mediated communication. J. Commun. Healthc..

[19] Ang C.S., Talib M.A., Tan K.A., Tan J.P., Yaacob S.N. (2015). Understanding computer-mediated communication attributes and life satisfaction from the perspectives of uses and gratifications and self-determination. Comput. Hum. Behav..

[20] Lewandowski J., Rosenberg B.D., Parks M.J., Siegel J.T. (2011). The effect of informal social support: Face-to-face versus computer-mediated communication. Comput. Hum. Behav..

[21] Bae M. (2016). The effects of anonymity on computer-mediated communication: The case of independent versus interdependent self-construal influence. Comput. Hum. Behav..

[22] Rains S.A. (2010). A meta-analysis of research on formal computer-mediated support groups: Examining group characteristics and health outcomes. Hum. Commun. Res..

[23] Andreassen H.K., Trondsen M., Kummervold P.E., Gammon D., Hjortdahl P. (2006). Patients who use e-mediated communication with their doctor: New constructions of trust in the patient-doctor relationship. Qual. Health Res..

[24] Ho S.M., Lowry P.B., Warkentin M., Yang Y., Hollister J.M. (2017). Gender deception in asynchronous online communication: A path analysis. Inf. Process. Manag..

[25] Adrianson L. (2001). Gender and computer-mediated communication: Group processes in problem solving. Comput. Hum. Behav..

[26] Wright K.B., Bell S.B. (2003). Health-related support groups on the Internet: Linking empirical findings to social support and computer-mediated communication theory. J. Health Psychol..

[27] Borowitz S.M., Wyatt J.C. (1998). The origin, content, and workload of e-mail consultations. JAMA.

[28] Colvin J., Chenoweth L., Bold M., Harding C. (2004). Caregivers of older adults: Advantages and disadvantages of Internet-based social support. Fam. Relat..

[29] Riordan M.A., Kreuz R.J. (2010). Emotion encoding and interpretation in computer-mediated communication: Reasons for use. Comput. Hum. Behav..

[30] Angeli C., Schwartz N.H. (2016). Differences in electronic exchanges in synchronous and asynchronous computer-mediated communication: The effect of culture as a mediating variable. Interact. Learn. Environ..

[31] Katz S.J., Moyer C.A., Cox D.T., Stern D.T. (2003). Effect of a Triage-based E-mail System on Clinic Resource Use and Patient and Physician Satisfaction in Primary Care: A Randomized Controlled Trial. J. Gen. Intern. Med..

[32] Joinson A.N. (2001). Self-disclosure in computer-mediated communication: The role of self-awareness and visual anonymity. Eur. J. Soc. Psychol..

[33] Patt M.R., Houston T.K., Jenckes M.W., Sands D.Z., Ford D.E. (2003). Doctors who are using e-mail with their patients: A qualitative exploration. J. Med. Internet Res..

[34] Tidwell L.C., Walther J.B. (2010). Computer-mediated communication effects on disclosure, impressions, and interpersonal evaluations: Getting to know one another a bit at a time. Hum. Commun. Res..

[35] Eveleigh R.M., Muskens E., van Ravesteijn H., van Dijk I., van Rijswijk E., Lucassen P. (2012). An overview of 19 instruments assessing the doctor-patient relationship: Different models or concepts are used. J. Clin. Epidemiol..

[36] Fuertes J.N., Anand P., Haggerty G., Kestenbaum M., Rosenblum G.C. (2015). The Physician–Patient Working Alliance and Patient Psychological Attachment, Adherence, Outcome Expectations, and Satisfaction in a Sample of Rheumatology Patients. Behav. Med..

[37] Teutsch C. (2003). Patient-doctor communication. Med. Clin. North Am..

[38] Ong L., De Haes J., Hoos A., Lammes F. (1995). Doctor-patient communication: A review of the literature. Soc. Sci. Med..

[39] Pian W., Song S., Zhang Y. (2020). Consumer health information needs: A systematic review of measures. Inf. Process. Manag..

[40] Dennis A., Fuller R., Valacich J. (2008). Media, tasks, and communication processes: A theory of media synchronicity. MIS Q. Manag. Inf. Syst..

[41] Liang B., Scammon D.L. (2011). E-Word-of-Mouth on health social networking sites: An opportunity for tailored health communication. J. Consum. Behav..

[42] Motamarri S., Akter S., Ray P., Tseng C.L. (2014). Distinguishing“ mHealth” from Other Healthcare Services in a Developing Country: A Study from the Service Quality Perspective. Commun. Assoc. Inf. Syst..

[43] Zhang X., Yan X., Cao X., Sun Y., Chen H., She J. (2018). The role of perceived e-health literacy in users’ continuance intention to use mobile healthcare applications: An exploratory empirical study in China. Inf. Technol. Dev..

[44] Kozinets R.V. (2002). The field behind the screen: Using netnography for marketing research in online communities. J. Mark. Res..

[45] Murthy D. (2008). Digital Ethnography: An Examination of the use of new technologies for social Research. Sociology.

[46] Analysys (2018). Annual Comprehensive Analysis of Internet Healthcare in China 2018. https://www.analysys.cn/article/analysis/detail/20018737.

[47] Burt J., Abel G., Elmore N., Lloyd C., Benson J., Sarson L., Carluccio A., Campbell J., Elliott M.N., Roland M. (2016). Understanding negative feedback from South Asian patients: An experimental vignette study. BMJ Open.

[48] Octacon (2018). Chinese Sentiment Analysis. https://github.com/octacon/bixin.

[49] Montini T., Noble A.A., Stelfox H.T. (2008). Content analysis of patient complaints. Int. J. Qual. Health Care.

[50] Liu C., Uffenheimer M., Nasseri Y., Cohen J., Ellenhorn J. (2019). “But His Yelp Reviews Are Awful!”: Analysis of General Surgeons’ Yelp Reviews. J. Med. Internet Res..

[51] Saldana J. (2009). The Coding Manual for Qualitative Researchers.

[52] Zhang X., Guo X., Lai K.-h., Yin C., Meng F. (2017). From offline healthcare to online health service: The role of offline healthcare satisfaction and habits. J. Electron. Commer. Res..

[53] Derks D., Fischer A.H., Bos A.E. (2008). The role of emotion in computer-mediated communication: A review. Comput. Hum. Behav..

[54] Guo S., Guo X., Fang Y., Vogel D. (2017). How Doctors Gain Social and Economic Returns in Online Health-Care Communities: A Professional Capital Perspective. J. Manag. Inf. Syst..

